# Immune Cell Signaling in Feline Infectious Peritonitis Virus Infection and Implications for Vaccine Design

**DOI:** 10.3390/vaccines14050435

**Published:** 2026-05-13

**Authors:** Hye-Mi Lee

**Affiliations:** College of Veterinary Medicine, Chungnam National University, Daejeon 34134, Republic of Korea; hyemi0728@gmail.com

**Keywords:** feline infectious peritonitis virus, vaccine design, immunopathogenesis, innate immune signaling, macrophages, antibody-dependent enhancement, immune regulation, adjuvants

## Abstract

Feline infectious peritonitis virus (FIPV) remains one of the most challenging viral diseases in veterinary medicine, largely owing to the absence of a consistently effective and safe vaccine. Despite widespread feline coronavirus infection, only a subset of infected cats progresses to feline infectious peritonitis, indicating that host immune responses are key determinants of disease outcomes. Accumulating evidence indicates that disease severity is driven not only by viral replication but also by macrophage- and monocyte-centered immune signaling, leading to excessive inflammation and systemic immunopathology in the host. Previous vaccine approaches against FIPV have failed to provide consistent protection and, in some cases, have been associated with enhanced disease. These outcomes suggest that vaccine-induced immune responses that recapitulate pathogenic signaling patterns may exacerbate disease rather than confer protection. In this review, we discuss the current knowledge of immune cell signaling pathways implicated in FIPV infection, including innate sensing through Toll-like receptors, downstream mitogen-activated protein kinases and NF-κB signaling, cytokine production profiles, Fc receptor-associated processes, and intracellular pathways such as autophagy, and how these mechanisms shape vaccine-induced immunity. By integrating insights from immune signaling kinetics, antibody functionality, adjuvant-driven pathway engagement, and platform-specific immune signatures, this review emphasizes the need to reframe FIPV vaccine development strategies that actively shape host immune responses. Rather than maximizing immunogenicity, successful vaccine design is likely to depend on limiting sustained macrophage activation and pro-inflammatory cytokine amplification while supporting antiviral immune functions, thereby reducing the risk of antibody-dependent enhancement and immunopathology. Beyond feline diseases, these considerations provide broader lessons for vaccine design in settings where immune-mediated pathology contributes to disease severity.

## 1. Introduction

Feline infectious peritonitis (FIP) remains one of the most challenging viral diseases in veterinary medicine because of the absence of a consistently effective and safe vaccine [1,2]. Although feline coronavirus infection is common in domestic cat populations, only a subset of infected animals develops FIP following the emergence of feline infectious peritonitis virus (FIPV) within the host [2,3]. This paradox indicates that host immune responses are key determinants of disease outcomes and represent a major obstacle to rational vaccine development [4].

Early experimental vaccine strategies against FIPV have shown limited efficacy and have failed to provide consistent protection [1,4]. In some experimental and clinical settings, vaccination has been associated with accelerated disease progression following viral challenge [5]. This phenomenon has been attributed, at least in part, to the antibody-dependent enhancement. (ADE) of infection, in which virus-specific antibodies facilitate viral entry into monocytes and macrophages [6,7]. These findings indicate that vaccine-induced immune responses may promote pathogenic mechanisms rather than protective immunity if immune signaling is not appropriately regulated [8,9]. Accordingly, the failure of previous vaccine approaches highlights the need to better understand the immune signaling pathways that shape disease outcomes during FIPV infection [8,9,10].

Several vaccine strategies, including inactivated whole-virus vaccines, recombinant spike-based approaches, and intranasal attenuated vaccines, have been evaluated for FIPV. Despite differences in antigen composition and delivery platforms, these approaches have failed to confer consistent protection and, in some cases, have been associated with disease enhancement. In these studies, these failures were not attributable to insufficient immunogenicity but were linked to ADE, macrophage infection, and dysregulated immune activation. A summary of the historical vaccine strategies and their associated immunological outcomes is presented in Table 1.

FIPV exhibits unique tropism for monocytes and macrophages, which are essential for innate immunity and antigen presentation [11,12]. Infection with these immune cells leads to dysregulated cytokine production and excessive inflammatory responses, driving systemic immunopathology [10,13]. The resulting inflammatory milieu contributes to the vascular damage, granulomatous lesions, and organ dysfunction characteristics of FIP [14]. As monocytes and macrophages are the primary targets of infection, the immune responses elicited during vaccination have the potential to modulate both viral replication and disease severity [9,12,15]. These observations suggest that immune signaling events occurring within infected monocytes and macrophages are critical determinants of whether immune activation results in protection or immunopathology.

Innate immune signaling pathways directly influence antiviral immunity by regulating cytokine production, antigen processing, and activation of adaptive immune responses [16,17]. Toll-like receptor (TLR) signaling and mitogen-activated protein kinase (MAPK) cascades are key components of these pathways that influence the magnitude and quality of immune activation following viral exposure [16,18]. In addition to surface and cytosolic sensing pathways, intracellular stress responses, such as autophagy, contribute to antigen processing and immune regulation during viral infection [19,20]. These signaling pathways not only influence antiviral defense but also determine whether immune activation is balanced or progresses toward pathological inflammation [21]. In the context of FIPV infection, dysregulation of innate immune signaling has been proposed to contribute to excessive inflammatory responses rather than effective viral control [9]. Accordingly, dissecting the innate immune signaling pathways engaged during infection provides an essential framework for understanding how vaccine-induced immune responses may be shaped toward protection rather than immunopathology [22].

Owing to the complex interplay between viral replication, immune cell signaling, and immunopathology, vaccine development for FIPV requires a framework that extends beyond antigen-centered approaches [8]. Accumulating evidence indicates that immune signaling pathways activated during infection influence not only disease progression but also the nature of the immune responses elicited by vaccination [22]. In particular, signaling events within monocytes and macrophages are critical determinants of whether immune activation results in protective immunity or pathological inflammation [2]. Accordingly, focusing on immune cell signaling provides an important perspective for understanding FIPV pathogenesis, which is highly relevant for the rational evaluation of vaccine-related strategies.

This review has three objectives. First, key innate immune signaling pathways are implicated in FIPV infection, with particular emphasis on monocytes and macrophages, which play a central role in FIP pathogenesis. Second, antibody-mediated mechanisms, including Fc receptor engagement and ADE, intersect with dysregulated immune responses in FIP. Third, critical considerations for FIP vaccine design include antigen selection, adjuvant choice, and vaccine platform-dependent immune signatures, with attention to immune regulation and disease-associated risk.

**Table 1:** Historical vaccine strategies against FIPV and associated immunological outcomes.

Vaccine Strategy	Target Antigen or Platform	Immune Response Characteristics	Experimental or Clinical Outcome	Key Immunological Limitation	Refs.
Inactivated whole-virus vaccine	Whole FIPV	Predominantly humoral responses reported in experimental settings	Induced humoral responses but failed to provide consistent protection against viral challenge in experimental studies	Inadequate control of macrophage infection and risk of ADE	[1,23,24]
Recombinant spike protein vaccine	Spike glycoprotein	Induction of spike-specific antibody responses	Accelerated disease progression and enhanced susceptibility following viral challenge	Fc receptor-mediated viral entry into macrophages	[5,6,25]
DNA-based vaccine	Spike or spike fragments	Immune responses reported in experimental models	Partial or inconsistent protection against viral challenge across experimental models	Immune dysregulation and insufficient protection	[8,26]
Intranasal temperature-sensitive live vaccine	Attenuated FIPV	Induction of local mucosal immune responses	Partial protection with limited efficacy in seronegative animals	Age-dependent efficacy and inconsistent immune control	[1,2]
Experimental viral vector approaches	Coronavirus antigens	Increased immunogenicity reported	Enhanced immunogenicity without consistent protection against disease progression	Potential risk of excessive innate immune activation	[2,9]

Reported experimental outcomes may vary depending on differences in study design, including the viral challenge dose, age, breed, serological status, and other experimental conditions.

## 2. Literature Search Strategy

This article was prepared as a narrative review, focusing on host immune signaling mechanisms during FIPV infection and their relevance to vaccine development. Relevant studies published until 2025 were identified through searches of the PubMed database using combinations of keywords, including FIPV, innate immunity, adaptive immunity, macrophages, monocytes, cytokines, ADE, and vaccine response. Original research articles and review papers relevant to FIPV immunopathogenesis and vaccine-associated immune responses were included. Studies not directly related to FIPV or vaccine-associated immune mechanisms were excluded. The selected literature was reviewed to summarize the current understanding of immune signaling pathways and their implications for rational vaccine design.

## 3. Immunopathogenesis of FIP as a Barrier to Vaccine Development

### 3.1. Protective Immunity vs. Pathogenic Immunity

Protective immunity against viral infection is generally defined as an immune response that restricts viral replication while maintaining tissue homeostasis [17,21,22]. In contrast, pathogenic immunity in FIPV infection refers to an immune response that fails to effectively control viral replication and instead contributes to disease progression through excessive inflammation, persistent monocyte and macrophage activation, and immune-mediated tissue injury [9,23,24]. However, in FIPV infection, immune activation does not uniformly correlate with protection, and disease progression is more often associated with dysregulated immune responses than with insufficient immunity [9]. Studies of naturally and experimentally infected cats have demonstrated that severe FIP is associated with elevated levels of pro-inflammatory cytokines, including tumor necrosis factor-α (TNF-α), interleukin-6 (IL-6), and interferon-γ (IFN-γ), particularly in the serum and affected tissues, consistent with the heightened activation of monocytes and macrophages and limited effective viral clearance [10,14,27].

Clinical and experimental observations have shown that cats with robust virus-specific immune responses are not necessarily protected from FIP [4,8]. Increased immunostimulant levels do not necessarily indicate protective immunity during FIPV infection, as excessive or dysregulated immune activation may contribute to inflammatory pathology and disease progression [1,2,28,29]. Accordingly, the qualitative characteristics and regulation of immune responses may be more relevant to protective outcomes than the magnitude of immune activation. Several studies have shown that cats with high titers of virus-specific antibodies or pronounced inflammatory responses can progress to fatal diseases, indicating that the magnitude of the immune response alone does not reliably predict disease progression and may coincide with pathogenic immune activation under certain conditions [5,6,8].

This paradox illustrates the distinction between protective and pathogenic immunity in FIP cats [8]. Immune responses that fail to effectively limit viral replication within target cells while promoting excessive inflammatory signaling are unlikely to confer protection and may exacerbate tissue damage [10]. Accordingly, protection against FIPV infection appears to depend on immune regulation and cellular targeting rather than on immune intensity alone. Consequently, the qualitative nature of the immune response appears to be a key determinant of disease outcome, independent of response magnitude alone [8]. In FIP, pathogenic immunity is characterized by sustained infection and activation of monocytes and macrophages rather than by the absence of an antiviral response, as these cells are the primary cellular targets of FIPV [9,12,30]. Infected macrophages have been shown to produce excessive levels of pro-inflammatory mediators, including TNF-α, IL-6, and granulocyte–macrophage colony-stimulating factor (GM-CSF), which amplify local and systemic inflammatory responses without effectively restricting viral replication [10,14,27]. This macrophage-driven inflammatory environment contributes directly to vasculitis, granulomatous lesions, and multi-organ involvement, which are the pathological hallmarks of FIP [9,13,28]. These inflammatory processes facilitate viral dissemination by promoting the survival, activation, and trafficking of infected immune cells, creating a feed-forward loop in which immune activation and viral persistence reinforce each other [9,28].

This distinction is particularly important [22]. Vaccination strategies that elicit strong immune activation in the absence of appropriate regulatory control may favor immunopathological mechanisms of protective immunity [5,26]. Accordingly, vaccine strategies that minimize excessive monocyte and macrophage activation while promoting coordinated antiviral immunity may be more favorable for reducing immunopathological outcomes during FIPV infections. Therefore, defining the features that distinguish protective immune responses from pathogenic ones is essential for the rational design of safe and effective vaccines against FIPV [8]. The distinct immunological features associated with protective and pathogenic immune responses during FIPV infection are summarized in Table 2.

### 3.2. Macrophage Infection and Antibody-Mediated Enhancement

A defining feature of FIPV infection is its tropism for monocytes and macrophages, which serve as the primary sites of viral replication and are key regulators of inflammatory responses [9,12,15,30]. Macrophage infection enables systemic viral dissemination and amplifies inflammatory responses that contribute to tissue injury [9,15,27].

The susceptibility of macrophages to infection has clear implications for humoral immunity [5,23]. In ADE, virus-specific antibodies facilitate viral entry into Fc receptor-expressing macrophages, resulting in increased intracellular infection instead of neutralization [5,23,34]. Antibodies directed against viral structural proteins, particularly spike glycoproteins, have been shown to enhance the infection of feline macrophages through Fc receptor-mediated uptake during FIPV infection [6,23,25]. These antibody-dependent mechanisms are associated with disease exacerbation rather than protective immunity [5,25].

Experimental vaccination and challenge studies have provided evidence that pre-existing antiviral antibodies can accelerate disease progression following FIPV challenge [5,24]. These observations indicate that antibody responses, when improperly directed or regulated, may contribute to enhanced macrophage infection and inflammatory pathology instead of protection [23,25,35].

This phenomenon highlights a fundamental challenge in FIPV vaccine development [5,9,25]. Antibody responses that fail to prevent macrophage infection may inadvertently promote pathogenic immune mechanisms, indicating the need to evaluate vaccine-induced humoral immunity beyond antibody titers [23,25,33]. Accordingly, the assessment of antibody specificity, functional activity, and interactions with macrophage-associated Fc receptor pathways is critical for the rational design of safe and effective vaccines against FIPV.

### 3.3. Immune Signaling Pathways Driving Immunopathology

The progression of FIP is closely linked to dysregulated immune signaling rather than uncontrolled viral replication alone [8,9]. Monocyte and macrophage infections initiate a cascade of intracellular signaling events that shape inflammatory responses and influence the balance between antiviral defence and tissue damage [9,28]. During FIPV infection, the activation of innate immune signaling pathways, including TLR-associated pathways involved in viral RNA sensing, is associated with the excessive production of pro-inflammatory mediators [9,10,27]. While these responses reflect the engagement of host defense mechanisms, their magnitude and persistence appear to favor systemic inflammation over effective viral control [8,32]. This imbalance contributes to the immunopathological features characteristic of FIP [8,9].

Innate immune sensing pathways and downstream signaling networks regulate cytokine expression, cell activation, and survival of infected immune cells [16]. When these pathways are inadequately regulated, inflammatory signaling may be amplified within monocytes and macrophages through pathways such as MAPK-dependent signaling, further enhancing viral replication and promoting its dissemination to multiple tissues [18,36]. Such sustained immune activation creates a feed-forward loop in which inflammation and viral persistence reinforce one another [8,27].

Immune signaling pathways that drive inflammation also influence antigen processing and the quality of adaptive immune responses [16,19]. Dysregulated signaling within antigen-presenting cells, particularly monocytes and macrophages, may alter the context in which viral antigens are present, shaping downstream immune responses toward pathogenic rather than protective outcomes [8,19]. This mechanism provides a link between immune signaling during infection and the failure of vaccine-induced immunity [8,22]. Several structural and accessory proteins of FIPV have been implicated in the modulation of host immune signaling pathways associated with inflammatory responses and viral persistence. In particular, the spike protein has been closely linked to viral entry into monocytes and macrophages and has been associated with ADE in experimental vaccine studies [5,35]. In addition, the accessory and non-structural proteins of feline coronaviruses have been proposed to influence intracellular signaling pathways and host immune responses, although the mechanistic characterization of these interactions remains limited [2,25]. These observations suggest that viral proteins involved in immune modulation may be important considerations during antigen selection and vaccine design against FIPV.

FIPV infection is also associated with several mechanisms that may contribute to the evasion of host immune responses [2,9]. The preferential infection of monocytes and macrophages may allow viral dissemination while simultaneously altering antigen presentation and inflammatory signaling within the infected immune cells [2,15]. In addition, sustained inflammatory activation and dysregulated cytokine production may impair the development of coordinated antiviral immune responses during disease progression [28,29,33]. These mechanisms may contribute to viral persistence and represent additional challenges for vaccine strategies aimed at inducing durable protective immunity against FIPV [2,3,35].

**Figure 1:**
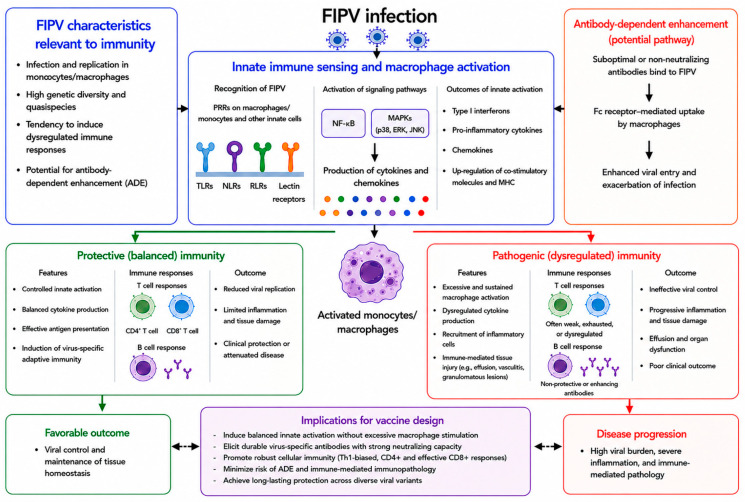
Proposed model of immune mechanisms and outcomes during FIPV infection and their implications for vaccine development. FIPV infects monocytes/macrophages and activates innate immune signaling through PRRs, leading to the production of cytokines and chemokines. Depending on the magnitude and regulation of these responses, infection results in either protective immunity with viral control or pathogenic immunity, characterized by excessive inflammation, ineffective viral control, and immune-mediated tissue damage. ADE may further exacerbate infections by facilitating Fc receptor-mediated viral entry into macrophages. An effective vaccine should induce balanced antiviral immunity while minimizing the risk of ADE and immunopathology. ADE, antibody-dependent enhancement; TLR, Toll-like receptor; MAPK, mitogen-activated protein kinase. Blue indicates innate immune activation, green indicates protective immunity, red indicates pathogenic immunity and disease progression, and purple indicates implications for vaccine design.The arrows indicate the proposed immunological relationships and progression pathways.

Together, these observations suggest that the immune signaling pathways activated during FIPV infection are the major drivers of immunopathology. Rather than representing the secondary consequences of infection, dysregulated signaling within monocytes and macrophages appears to be a primary determinant of disease severity. During vaccine development, these signaling pathways may represent critical immunological barriers that must be addressed to avoid recapitulating pathogenic immune trajectories after vaccination (Figure 1).

### 3.4. Adaptive Immune Responses and Implications for Vaccine Development

Adaptive immune responses contribute to both viral control and immunopathological outcomes of FIPV infection. Although the protective correlates of immunity remain incompletely defined, available evidence suggests that effective cellular immune responses are associated with resistance to disease progression in some cats [8,37]. Experimental and clinical observations indicate that cats that do not develop FIP following FCoV exposure often exhibit stronger virus-specific cellular immune responses, including IFN-γ-associated T cell activity, than those that develop clinical disease [37,38]. These findings suggest that the quality and regulation of adaptive immunity, rather than the magnitude of immune activation alone, may influence the outcome of FIPV infection [2].

Humoral immune responses also appear to have context-dependent roles in FIPV infections. While virus-specific antibodies may contribute to viral neutralization under certain conditions, antibody responses have also been implicated in ADE through Fc receptor-mediated uptake of virus-antibody complexes by monocytes and macrophages [23,25]. This mechanism has been proposed as an explanation for the inconsistent or pathogenic outcomes observed in several historical vaccine studies targeting the FIPV spike protein [5,35]. Accordingly, antibody responses during FIPV infection may contribute to either antiviral protection or enhanced inflammatory pathology, depending on antibody specificity, functional activity, and the immunological context in which these responses are generated [2].

Together, these observations suggest that successful vaccine strategies against FIPV will likely require a balanced induction of antiviral adaptive immunity while limiting excessive macrophage-associated inflammatory responses. Vaccine approaches that promote durable cellular immune responses without enhancing pathogenic inflammatory signaling may therefore be more effective in preventing disease progression and immunopathology following viral exposure [2,25].

## 4. Innate Immune Signaling Pathways Shaping Vaccine Responses in FIPV

### 4.1. TLR Signaling

TLRs play a key role in the innate immune recognition of viral infections by sensing conserved pathogen-associated molecular patterns and initiating downstream inflammatory signaling [39]. Activation of TLR pathways induces cytokine and chemokine production, which shapes the magnitude and quality of subsequent immune responses and influences both antiviral defense and immunopathology [39].

In FIPV infections, innate immune activation is prominent within infected monocytes and macrophages, which express multiple TLR family members and serve as the primary targets for viral replication [9,10,40]. The engagement of TLR-associated signaling in these cells contributes to the production of pro-inflammatory mediators observed during the disease, consistent with an attempt to control infection while simultaneously shaping inflammatory outcomes [41]. As discussed in Section 3.3, sustained or dysregulated activation of these pathways favors systemic inflammation rather than effective viral control.

TLR signaling contributes to immune priming during vaccination but can also amplify inflammatory responses when excessively or inadequately regulated. While appropriate TLR engagement may support antigen presentation and adaptive immune activation, excessive or poorly regulated TLR-driven signaling can amplify inflammatory responses and contribute to immunopathology [9,21]. This balance is particularly relevant for FIPV, in which macrophage-driven inflammation is a key feature of disease pathogenesis. Therefore, effective vaccine strategies may require balanced immune activation that supports antiviral immune responses while limiting excessive macrophage-associated inflammatory signaling. The immune cell signaling pathways implicated in FIPV pathogenesis and their implications for vaccine responses are summarized in Table 3.

Accordingly, vaccine strategies that rely on TLR engagement, including adjuvant-based approaches, must consider the intensity, timing, and cellular context of innate immune stimulation. Instead of maximizing innate activation, effective FIPV vaccines are likely to require controlled TLR signaling that promotes immune priming without recapitulating the inflammatory pathways associated with disease progression. Understanding how TLR pathways are engaged and regulated during FIPV infection provides a framework for evaluating adjuvant selection and vaccine-induced immune responses.

### 4.2. MAPK Pathways

In FIP, the activation of intracellular signaling pathways downstream of innate immune receptors contributes to macrophage activation and the production of inflammatory mediators. The MAPK pathway functions as an intracellular signaling module that integrates signals from innate immune receptors and regulates the expression of genes associated with inflammation, cell survival, and stress responses [18]. Activation of MAPK cascades, including ERK, JNK, and p38 pathways, is commonly observed during immune cell activation following viral infection [42].

Although direct mechanistic studies of MAPK signaling in FIPV infection remain limited, inflammatory signaling downstream of innate immune receptors in monocytes and macrophages is consistent with the cytokine profiles and activation states observed during disease progression [9,33,36]. In these cells, MAPK signaling contributes to the transcriptional regulation of pro-inflammatory mediators and influences both antiviral responses and tissue-damaging inflammation.

MAPK pathways are relevant for vaccine development because they act downstream of multiple innate immune sensors, including TLRs, and modulate the strength and duration of immune activation [22]. Vaccine formulations that strongly engage MAPK signaling may enhance immune responses but also risk amplifying inflammatory pathways when regulatory mechanisms are not adequately engaged [18,36]. Accordingly, MAPK signaling functions as an intracellular checkpoint that determines whether vaccine-induced innate activation remains protective or progresses to immunopathology. Thus, considering MAPK-dependent signaling is important for understanding how vaccine-induced innate activation intersects with macrophage-driven inflammatory pathways characteristic of FIPV infection.

### 4.3. Cytokine Balance and Immunopathology

Cytokine production is a key effector mechanism through which innate immune signaling pathways translate into inflammatory pathology during FIP [9,10]. Instead of reflecting a coordinated antiviral response, disease progression is associated with dysregulated cytokine expression patterns that amplify inflammation and tissue damage.

Multiple studies have reported elevated levels of pro-inflammatory cytokines, including TNF-α, IL-6, and IFN-γ, in the serum and lesions of cats with FIP, linking cytokine imbalance to disease severity and systemic inflammation [10,13,27]. These mediators are primarily produced by activated monocytes and macrophages and contribute to vascular dysfunction, leukocyte recruitment, and granulomatous lesion formation, which are characteristic of advanced disease [9,43].

Cytokine-driven inflammation in FIPV infections is not adequately counterbalanced by regulatory mechanisms. Excessive production of pro-inflammatory cytokines promotes macrophage survival and activation, creating a self-sustaining inflammatory environment that supports continued viral persistence [9,14]. This imbalance between inflammatory and regulatory signals reinforces immunopathology rather than facilitating effective viral clearance.

Cytokine regulation is an important factor in vaccine development. Vaccine-induced immune responses that disproportionately favor pro-inflammatory cytokine production may recapitulate the cytokine profiles associated with disease progression. Conversely, immune responses that promote controlled cytokine signaling may support antiviral defense while limiting the tissue damage. Together, these observations indicate that successful FIPV vaccines must not only elicit antiviral immunity but also maintain an appropriate cytokine balance. Avoiding excessive or prolonged inflammatory cytokine responses is essential for preventing immunopathological outcomes following vaccination.

**Table 3:** Immune cell signaling pathways are implicated in FIPV pathogenesis.

Signaling Pathway	Primary Cell Type Involved	Key Downstream Effects	Contribution to Disease Progression	Implications for Vaccine Responses	Refs.
TLR signaling	Monocytes, macrophages	Induction of inflammatory cytokines and chemokines	Amplification of macrophage-driven inflammatory responses	Excessive innate stimulation may favor pathological immune activation	[9,16,32]
MAPK pathways	Macrophages	Transcriptional regulation of inflammatory mediators	Sustained inflammatory signaling associated with tissue damage	Strong MAPK activation may increase immunopathological risk	[18,33,36]
Fc receptor-mediated signaling	Monocytes, macrophages	Fc-dependent viral uptake and intracellular signaling	ADE and viral dissemination	Antibody quality rather than magnitude determines safety	[5,23,25]
Cytokine-driven feedback loops	Infected immune cells	Persistent production of pro-inflammatory cytokines	Feed-forward inflammation and immune dysregulation	Balanced cytokine induction is required for protective immunity	[9,21,27]
Autophagy-associated signaling	Antigen-presenting cells	Modulation of intracellular antigen processing	Potential immune skewing through altered antigen presentation	Intracellular pathways may shape vaccine-induced immunity	[19,44,45]

TLR, Toll-like receptor; MAPK, mitogen-activated protein kinase; ADE, antibody-dependent enhancement.

## 5. Autophagy and Intracellular Pathways as Modulators of Vaccine-Induced Immunity

### 5.1. Autophagy in Antigen Processing

Autophagy is a conserved intracellular degradation pathway that contributes to cellular homeostasis and host defense by delivering cytoplasmic components to lysosomes [46]. In immune cells, including monocytes, macrophages, and dendritic cells, autophagy contributes to antigen processing and presentation by regulating the availability and intracellular routing of endogenous antigens and influencing major histocompatibility complex-mediated pathways [47,48].

Through the sequestration and degradation of cytosolic proteins and pathogen-derived components, autophagy facilitates the generation of pro-inflammatory peptides that are subsequently presented to T cells [49,50]. This function provides a mechanistic link between innate and adaptive immune activation in professional antigen-presenting cells, indicating that autophagy acts as a modulatory component of immune regulation rather than as a primary effector pathway [19].

During vaccination, autophagy influences the efficiency and qualitative features of antigen presentation [19,44]. Vaccine antigens that gain access to autophagic pathways may be processed differently from those restricted to endosomal compartments, and may shape the specificity and functional polarization of T cell responses [44]. Accordingly, autophagy contributes to the regulation of vaccine-induced immune quality, independent of immune magnitude, by modulating intracellular antigen processing and presentation.

### 5.2. Potential Interactions Between FIPV and Autophagy Pathways

Many viruses interact with host autophagy pathways to support specific stages of their life cycle or modulate host immune responses [19,51]. Depending on the virus and cellular context, these interactions can result in the activation, inhibition, or subversion of autophagy [52].

Currently, direct mechanistic evidence supporting the role of autophagy in FIPV replication remains limited [45,53]. However, FIPV infection of monocytes and macrophages is associated with cellular stress responses and inflammatory signaling pathways that intersect with autophagy-related processes described in other viral systems [19,53,54]. In this setting, autophagy is more likely to be engaged as part of a generalized cellular stress and immune response than as a virus-specific replication strategy, providing a plausible framework for autophagy activation during infection.

Instead of functioning solely as an antiviral defense mechanism, autophagy in infected immune cells may influence the intracellular environment, affecting viral persistence, immune activation, and cell survival [19,54]. These effects are likely to occur indirectly through the modulation of macrophage function and inflammatory signaling rather than through direct manipulation of the autophagic machinery by FIPV. Consequently, autophagy may contribute to disease outcomes by shaping immune cell behavior during FIPV infection, even in the absence of direct viral control of autophagic pathways.

### 5.3. Implications for Antigen Presentation

Autophagy-associated antigen processing influences the initiation and regulation of immune responses by shaping the availability and intracellular routing of antigens for presentation to T cells [47,48]. Through its effects on antigen processing, autophagy modulates T-cell priming and downstream immune polarization [48,49]. This regulatory function is particularly relevant in macrophages and other antigen-presenting cells that play key roles in FIP pathogenesis [9,43].

In vaccination settings, autophagy modulation may influence the qualitative features and durability of vaccine-induced immune responses [19,20,55]. Antigens that access autophagic pathways may be processed and presented differently from those restricted to endosomal routes and may promote balanced immune activation, whereas dysregulated autophagy may exacerbate inflammatory signaling in immune cells [19,44].

Since immune dysregulation plays a key role in FIP, considering autophagy-related pathways provides an additional framework for evaluating vaccine strategies [8,9]. Instead of serving as a passive consequence of antigen uptake, intracellular antigen processing represents a regulatory checkpoint that integrates antigen handling and innate immune signaling. Accordingly, integrating autophagy-associated antigen processing into vaccine design considerations can help guide the development of immune responses that favor protection while minimizing the risk of immunopathology.

## 6. Immunomodulatory Strategies and Host Targeting Approaches

### 6.1. Modulating Signaling Pathways

Evidence from FIP indicates that disease severity is shaped more by the magnitude, duration, and cellular context of host immune signaling than by viral replication alone [8,9]. In affected cats, macrophage and monocyte activation is accompanied by elevated levels of inflammatory mediators, such as TNF-α, IL-6, IFN-γ, and GM-CSF in the serum and lesions, supporting the observation that pathogenic outcomes can arise from sustained pro-inflammatory cytokine production rather than insufficient immune activation [10,14,27]. This distinction has direct implications for vaccine design, as vaccine-induced responses that recapitulate macrophage-centered inflammatory signaling patterns may increase the risk of immunopathology [5,24].

Innate sensing pathways that constitute antiviral immunity also act as drivers of pathology when their activation is excessive or insufficiently resolved [56]. In macrophages and monocytes, RNA sensing through TLR3, 7, and 9, together with cytosolic sensors that converge on NF-κB and interferon regulatory factor (IRF) 3 or 7, can promote transcriptional programs that include TNF-α and IL-6, as well as chemokines that recruit additional inflammatory cells [16,21,57,58]. These pathways are indispensable for immune priming; however, persistent activation within Fc receptor-expressing target cells may reinforce a feed-forward inflammatory state that supports tissue injury and systemic disease features [12,43]. Therefore, the key issue is not whether innate sensing occurs, but whether signaling is temporally constrained and counterbalanced by regulatory mechanisms, including IL-10-mediated control [59,60].

In FIPV infection, specific mutations within the spike protein are associated with a shift in viral tropism toward monocytes and macrophages, resulting in enhanced infection of Fc receptor-expressing target cells [15]. Such FIPV-specific observations link viral determinants to the sustained activation of macrophage-associated signaling pathways and provide a disease-relevant context in which dysregulated innate signaling can directly contribute to the immunopathology.

Downstream signaling modules further influence whether innate activation is protective or pathogenic. MAPK cascades, including ERK, JNK, and p38, regulate cytokine transcription and stress responses and can prolong the expression of inflammatory mediators when strongly engaged [18]. In addition, Fc receptor-associated signaling in macrophages can couple antibody binding to intracellular activation programs that modulate viral uptake and inflammatory output, providing a mechanistic link between humoral responses and macrophage-centered pathology in settings where antibody-dependent enhancement is possible [5,61]. These considerations emphasize that vaccine-induced immune activation should be evaluated not only by response magnitude but also by signaling kinetics and cellular targeting within monocytes and macrophages, which represent the primary cellular compartment determining FIP outcomes.

This reframes immune signaling as a design variable that can be tuned through dose, route, antigen delivery context, and adjuvant-driven pathway engagement to support antigen presentation and antiviral priming while limiting sustained TNF-α- and IL-6-dominated signaling trajectories in macrophage and monocyte populations [62,63].

### 6.2. Host-Directed Adjuvants

Experimental vaccine studies on feline coronavirus infections have demonstrated that the qualitative nature of the host immune response strongly influences the disease outcome [8,9]. Early vaccination approaches using viral vectors or whole-virus platforms showed that enhanced immune activation did not uniformly confer protection and, in some cases, was associated with accelerated disease following the challenge [5,24]. These observations indicate that immune amplification alone is insufficient, and in FIPV, it can exacerbate the pathogenic processes [2].

Subsequent studies have shown that the host immune context at the time of antigen exposure critically shapes the vaccine outcomes [8,13,60]. In peptide-based FIPV vaccine models, the inclusion of CpG oligodeoxynucleotides (ODN) altered immune polarization, but protective efficacy depended on the dose, immunization schedule, and the resulting balance between cellular and humoral responses [64]. These findings indicate that the adjuvant effects of FIPV are context-dependent and that inappropriate immune skewing may negate its potential benefits [8,64].

Beyond adjuvant composition, differences in immune profiles have been observed even when identical viral antigens are used [60]. Analyses of cytokine expression and inflammatory signatures in experimentally infected cats have revealed that variations in host response patterns, rather than viral factors alone, are associated with divergent clinical outcomes [13,65]. These data support the view that modulating host immune trajectories is a relevant intervention point for FIPV vaccine design [9].

Together, these studies suggest that host-directed adjuvant strategies for FIPV should not be viewed as a search for stronger immune stimulation [8,28]. Instead, they emphasized the importance of shaping immune responses compatible with the immunopathological constraints of the disease [28]. This perspective necessitates evaluation frameworks that extend beyond antibody titers to include indicators of inflammatory balance and myeloid activation and align vaccine assessment with the mechanisms that drive disease progression [25,65].

### 6.3. Lessons from Antiviral Therapies

Experience with antiviral therapies for FIP has provided insights into the dissociation between viral replication and immune-mediated disease progression [2]. Treatment studies using direct-acting antivirals, including nucleoside analogs such as GS-441524 and 3C-like protease inhibitors, have shown that rapid suppression of viral replication is frequently accompanied by clinical improvement and increased survival, even in cats with advanced diseases [66,67].

However, large-scale clinical analyses of cats treated with GS-441524 have revealed the limitations of antiviral therapy, including variable treatment responses and incomplete recovery in subsets of cats with advanced or neurological disease [68]. While viral load reduction leads to the resolution of fever, effusions, and systemic signs in many cases, inflammatory lesions and neurological manifestations may persist or recover incompletely, particularly when treatment is initiated after extensive immune-mediated tissue damage [69,70]. These findings indicate that once inflammatory cascades and macrophage-driven pathology are established, antiviral control alone may be insufficient to reverse the disease-associated immune alterations [28].

This divergence illustrates a fundamental distinction between therapeutic and prophylactic interventions against FIPV [8]. Antiviral therapies act downstream of infection, targeting viral replication after the initiation of pathogenic immune responses [2]. In contrast, effective vaccines must function upstream to prevent the emergence of maladaptive immune trajectories that underlie disease progression. Consequently, vaccine strategies evaluated solely on their ability to suppress viral replication risk overlook the host-driven mechanisms that ultimately determine disease severity [5,25].

Lessons from antiviral treatment studies indicate that successful vaccine development for FIPV requires an integrated approach that combines viral control with the modulation of host immune responses [2]. Preventing the initiation of pathogenic immune activation, rather than attempting to correct established inflammatory states after disease onset, represents a central design principle for future prophylactic strategies [2].

## 7. Implications for Rational Vaccine Design Against FIPV

### 7.1. Antigen Selection

Antigen selection is a key determinant of vaccine outcomes in FIPV infections, as immune recognition of viral components does not uniformly translate into protection [8,9]. In FIPV, antigen-specific immune responses can intersect with host pathways that drive disease progression, indicating the need to evaluate not only whether an antigen is immunogenic, but also how antigen-induced responses engage immune effector mechanisms implicated in pathology [2,28].

The viral spike protein exemplifies this challenge. Spikes are essential for viral entry and are the dominant targets of neutralizing antibodies in coronaviruses [71,72]. However, in FIPV, spike-directed antibody responses have been directly implicated in ADE and macrophage-mediated disease exacerbation, particularly through Fc receptor-dependent uptake of virus–antibody complexes [5,23]. These observations indicate that neutralizing activity alone is insufficient to define protective immunity against FIPV and that the functional properties of antibody responses, including Fc-mediated effector engagement, are determinants of the outcome [8,25].

Insights from broader coronavirus vaccine research support this perspective, showing that antibodies with comparable neutralizing capacities can differ substantially in their downstream immunological consequences [73,74,75]. Antibody subclass, epitope specificity, and conformational recognition have been shown to influence Fc receptor engagement and inflammatory signaling and shape the balance between protection and immunopathology [8,9]. These findings are particularly relevant for FIPV, in which macrophage-centered immune activation is the key pathogenic mechanism [13,60].

Together, these data indicate that rational antigen selection for FIPV should move beyond whole-protein immunodominance toward designs that decouple viral neutralization from pathogenic Fc receptor-mediated processes [2]. Strategies that focus on defined antigen subdomains, conformationally constrained epitopes, or spike regions with a reduced propensity to drive Fc-dependent uptake may preserve protective recognition while limiting the engagement of disease-associated immune pathways [75,76,77]. Incorporating antigens that preferentially elicit T cell-mediated immunity may counterbalance antibody-driven mechanisms that carry inherent risks in FIPV [8,15].

Therefore, antigen selection for FIPV vaccines should not be viewed as a static choice of viral components but as a strategic process informed by concepts of antibody functionality and immune modulation. Within this framework, antigen design is the primary lever for shaping vaccine efficacy and safety.

### 7.2. Adjuvant Choice

In FIPV vaccine development, the choice of adjuvant should be guided by disease-specific immunological constraints rather than general measures of immunogenicity [8,9]. Because macrophage activation and dysregulated innate responses are key features of FIPV pathogenesis, adjuvants that strongly amplify myeloid inflammatory signaling require particular caution [13,60].

Classical innate-stimulating adjuvants, such as aluminum salts and oil-in-water emulsions, have been widely used to enhance antibody responses in many vaccine platforms [63,78]. However, these formulations are known to promote robust innate activation and inflammatory cytokine induction, properties that may be poorly aligned with the macrophage-centered immunopathology of FIPV [2,62]. In this context, indiscriminate enhancement of innate signaling could recreate the immune environment associated with disease progression [5].

Similarly, Toll-like receptor agonists, including CpG ODN, illustrate the context-dependent nature of adjuvant effects in FIPV [64]. While CpG-based formulations can bias immune responses toward cellular immunity, experimental studies on feline coronavirus systems have shown that immune outcomes vary substantially depending on the dose and immunization schedule [8,64]. These findings indicate that innate bias alone is insufficient to predict safety or efficacy, and that excessive or poorly controlled stimulation may negate potential benefits.

Adjuvants that induce sustained inflammatory cytokine production in monocytes and macrophages are potential risk factors for FIPV [9,28]. In contrast, adjuvant strategies that support antigen presentation and immune priming without prolonged inflammatory amplification are more compatible with the disease’s pathological features [79]. Thus, the controllability of innate activation, duration of signaling, and extent of macrophage engagement are the criteria for adjuvant selection.

Accordingly, the evaluation of adjuvant performance in FIPV should extend beyond antibody titers or response magnitude [25,80]. The assessment of cytokine profiles, macrophage activation states, and early innate response dynamics provides a disease-relevant framework for comparing adjuvant candidates [28,65]. Within this framework, adjuvants are best viewed not simply as immune enhancers, but also as determinants of vaccine-induced immune trajectories.

### 7.3. Avoiding ADE and Pathogenic Immune Activation

Avoiding ADE and pathogenic immune activation remains a key challenge for vaccines targeting FIPV [8]. Historical vaccination experiments have shown that immune responses directed toward viral antigens can accelerate disease following a challenge rather than confer protection, including early death observed after immunization with a recombinant vaccinia virus expressing the spike protein [5].

Mechanistic studies have further clarified that the antibody-dependent enhancement of FIPV is mediated by antibody–virus complexes that promote the infection of Fc receptor-expressing monocytes and macrophages [23]. In vitro studies using monoclonal antibodies against the spike protein showed that antibodies can enhance the infection of primary feline macrophages, and that enhancement can occur even with antibodies that display neutralizing activity [23,35]. Antibody characteristics influence enhancement, as IgG subclass differences have been shown to alter the magnitude of enhancement in feline macrophage models [81]. In addition, sera from experimentally or naturally infected cats can mediate the enhancement of macrophages, supporting the biological relevance of this mechanism beyond monoclonal antibody systems [6].

Although macrophage infection and Fc receptor-associated immune activation are directly supported by studies on FIPV and feline coronavirus, several principles regarding antibody quality, Fc functionality, and vaccine platform-dependent immune signatures have been derived from studies on other coronavirus systems [8]. Recent research on coronavirus systems has refined the interpretation of the enhancement risk by emphasizing epitope specificity, affinity, and Fc-mediated effector engagement, rather than antibody quantity alone [75]. These factors can shape inflammatory signaling and myeloid cell responses in ways that are not captured by neutralization assays and provide a framework for interpreting vaccine-induced humoral immunity against FIPV, where macrophage tropism and Fc receptor-mediated uptake are linked to viral replication and inflammatory amplification [9,23,73,74].

For rational vaccine design, minimizing the risk of enhancement requires the active consideration of how vaccine-induced antibodies interact with Fc receptor-expressing cells [73,74]. Antigen designs that reduce the induction of non-neutralizing antibody responses, together with vaccine strategies that support cellular immune responses without amplifying macrophage-centered inflammation, may reduce the likelihood of pathogenic immune activation [8,75]. Accordingly, the avoidance of ADE should be regarded as a design principle for FIPV vaccines, and candidate vaccines should be evaluated not only for antiviral immunity but also for cytokine and signaling profiles associated with the disease [5,35]. The results are presented in Table 4.

In addition to innate and antibody-mediated mechanisms, T-cell responses represent an underemphasized component of protective immunity against FIP. Studies in naturally infected cats have reported alterations in T cell populations and functions, including reduced peripheral T cell numbers and impaired cell-mediated immunity in cats that progress to FIP, suggesting that deficient or dysfunctional T cell responses contribute to disease susceptibility [8,9]. For vaccine development, these observations indicate that effective FIP vaccines should not only avoid excessive macrophage-centered inflammatory signaling but also support functional T cell priming, particularly Th1-associated responses that promote antiviral activity without exacerbating immunopathology. Accordingly, vaccine platforms and adjuvant strategies that favor coordinated CD4+ and CD8+ T-cell responses can provide an additional layer of protection by complementing humoral immunity and restraining antibody-driven disease mechanisms.

## 8. Future Perspectives and Challenges

### 8.1. Translational Gaps

Despite extensive experimental research on FIPV, the translation of mechanistic insights into effective and reliable vaccines remains limited [2,8]. This limitation does not primarily reflect a lack of immunological knowledge but rather persistent gaps between experimental model systems and the complex immune environment encountered during natural infections [9,28].

A key challenge arises from the reliance on controlled in vitro systems and experimental challenge models, which inherently reduce biological variability [8,9,60]. While such approaches have been instrumental in defining viral entry mechanisms, immune activation pathways, and vaccine-induced responses, they often fail to capture the heterogeneity of the immune states present in naturally exposed outbred feline populations [60]. Age, prior coronavirus exposure, genetic background, and environmental factors collectively shape immune responsiveness; however, these variables are rarely incorporated into traditional FIPV vaccine evaluation pipelines [1,8].

Recent advances in systems immunology and vaccinology indicate that vaccine outcomes are strongly influenced by pre-existing immune landscapes rather than antigen-specific responses alone [86,87,88]. Under experimental conditions, immune responses that appear protective may rely on narrowly defined activation thresholds that do not generalize to real-world settings [86]. In FIPV, where disease susceptibility is closely linked to immune regulation rather than immune magnitude, this discrepancy may contribute to the repeated failure of promising vaccine candidates during translational progression [8,28].

Bridging this gap will require integrative strategies that explicitly connect mechanistic studies with immune profiling in naturally infected or vaccinated animals [86,87]. Longitudinal monitoring of immune parameters, combined with system-level analyses, offers a path toward identifying immune configurations associated with protection against pathogenic activation [86,87,88]. Although such approaches are increasingly applied in human vaccinology, they remain underutilized in FIPV research [86,87,88]. Incorporating these frameworks into future vaccine development efforts may be essential for overcoming long-standing translational barriers in this field [79,80].

### 8.2. Safety Considerations

Safety remains a major barrier to the successful development of vaccines against FIPV [8]. Unlike many viral infections, in which adverse immune events are infrequent or secondary, immune-mediated pathology is a key driver of FIP [9,28]. Therefore, vaccine safety in this setting cannot be defined solely by the absence of acute toxicity or short-term effects after immunization [5].

Recent advances in vaccinology indicate that immune safety should be evaluated at the level of immune configuration rather than by clinical outcomes alone [62,79,83]. In FIPV, this necessitates a careful assessment of qualitative immune features, including antibody specificity and functionality, Fc receptor engagement, macrophage activation state, and cytokine balance, following vaccination [8,23]. Immune responses that appear immunogenic under conventional criteria may establish an inflammatory predisposition with latent pathogenic potential [9,28,80].

These considerations indicate the need for safety assessment frameworks tailored to immunopathological diseases [28]. For FIPV, preventing the induction of pathogenic immune trajectories is as important as achieving antiviral immunity [5,8]. Accordingly, future vaccine studies should integrate immune profiling into safety evaluations, recognizing that immune dysregulation may precede overt clinical diseases and may not be captured during short-term observation periods.

### 8.3. Platform-Based Vaccines

The emergence of platform-based vaccine technologies has enabled rapid and adaptable vaccine development for multiple viral systems [89,90]. Platforms such as mRNA, viral vectors, and nanoparticle-based formulations offer a high degree of control over antigen expression, presentation, and kinetics, creating opportunities to revisit longstanding challenges in FIPV vaccine design [90,91].

Evidence indicates that these platforms are not immunologically neutral [85,92,93,94]. Distinct platforms impose characteristic innate signaling profiles that influence antigen processing, immune cell recruitment, and downstream adaptive responses [92,93,94]. Platform-specific immune signatures are significant in diseases such as FIPV, where relatively subtle differences in innate activation can shift immune responses from protective to pathogenic [8,28].

One advantage of platform-based approaches is the partial decoupling of antigen design from immune modulation [90]. Platforms that allow precise adjustment of antigen dose, temporal expression, or innate signaling intensity can enable iterative optimization aimed at limiting excessive macrophage activation while preserving antiviral immunity [84,90]. Accordingly, the development of mRNA and other platform-based vaccine strategies against FIPV may require careful regulation of innate immune activation to support antiviral immunity while limiting excessive inflammatory signaling and macrophage-associated immunopathology. This flexibility aligns with the need for fine control over the immune trajectories identified in this review.

Simultaneously, platform-based vaccines introduce additional variables that complicate safety and translational assessments [22,79,94]. Differences in delivery kinetics, tissue distribution, and innate sensing pathways can generate immune configurations that are difficult to predict based on antigen properties alone [62,94]. In FIPV-specific immunopathology, these variables must be evaluated with caution, as platform-driven innate cues can recreate immune environments associated with disease progression [2,5].

Platform-based vaccines represent both an opportunity and challenge for FIPV [62]. Their successful application depends not only on technological novelty but also on the alignment between platform-induced immune signatures and the immunological constraints that define disease susceptibility in FIP.

## 9. Conclusions

FIPV infection exemplifies a disease in which host immune responses, rather than viral replication, are the key determinants of clinical outcomes. Across experimental, pathological, and clinical observations, the evidence reviewed here converges to the conclusion that dysregulated immune cell signaling within monocytes and macrophages underlies the inflammatory cascades and tissue injury characteristics of FIP. Innate signaling pathways, cytokine regulation, and Fc receptor-associated processes collectively shape the immune environment, which can restrain disease or promote immunopathology.

This immunological framework provides a unifying explanation for the persistent challenges encountered in FIPV vaccine development. Antigen selection studies have shown that immune recognition does not uniformly translate into protection, particularly when antibody responses engage Fc receptor-expressing cells and amplify macrophage activation. Adjuvant choice and platform-specific immune signatures are important variables, as these components actively shape innate activation patterns and downstream immune trajectories, rather than serving as neutral enhancers of immunogenicity. Experimental evidence of antibody-dependent enhancement and pathogenic immune activation further indicates that immune magnitude alone is an insufficient and potentially misleading metric of vaccine efficacy.

The consideration of translational gaps and safety constraints reinforces the need to evaluate vaccines within the context of immune regulation rather than short-term clinical endpoints. Controlled experimental models have been invaluable for defining mechanisms; however, they often fail to capture the heterogeneity of immune states encountered in naturally infected outbred feline populations. In FIPV, immune dysregulation may precede overt disease, indicating the importance of safety frameworks that assess immune configuration, durability, and balance in addition to antiviral activity.

By integrating insights from immune signaling, antibody functionality, adjuvant effects, and platform-based vaccine technologies, this review emphasizes that successful FIPV vaccination requires strategies that actively shape the immune regulation. Protection is likely to depend on the ability to promote balanced antiviral immunity while avoiding immune trajectories associated with macrophage-derived inflammation and excessive innate activation.

Beyond feline diseases, FIPV provides a well-defined example of the broader challenges faced in the development of vaccines for immunopathological viral infections. Viewing immune cell signaling as a primary determinant of outcomes offers a coherent framework for interpreting past vaccine failures and refining future vaccine design strategies, prioritizing immune quality, contextual safety, and translational relevance.

## Figures and Tables

**Table 2:** Distinct features of protective and pathogenic immunity in FIPV infection.

Immunological Feature	Protective Immune Response	Pathogenic Immune Response	Relevance to Vaccine Design	Refs.
Viral replication	Restricted or controlled	Persistent replication in macrophages	Vaccines must limit macrophage permissiveness	[8,9]
Antibody function	Antibody responses not associated with Fc receptor-mediated enhancement	Fc receptor-dependent viral entry	Antibody quality is critical	[5,23,31]
Cytokine profile	Balanced inflammatory signaling	Excessive pro-inflammatory cytokines	Avoid hyperinflammatory immune states	[21,32,33]
Macrophage activation	Regulated activation	Hyperactivation and dissemination	Control of innate signaling is essential	[9,27]
Clinical outcome	Absence of clinical FIP	Development of FIP	Safety is a primary endpoint	[1,2]

**Table 4:** Vaccine design considerations for immunopathological viral diseases, including FIPV.

Design Parameter	Conventional Vaccine Approach	Considerations Specific to FIPV	Supporting Rationale	Refs.
Antigen selection	Use of immunodominant viral proteins	Avoidance of epitopes associated with ADE	Neutralizing activity alone does not predict protective outcome in FIPV	[6,8,35]
Adjuvant choice	Strong innate immune stimulation	Controlled and balanced innate signaling	Excessive myeloid activation may promote immunopathology	[9,22,82]
Immune response metrics	Antibody titer magnitude	Quality and functional profile of immune responses	Traditional correlates may not capture enhancement or dysregulation	[25,74,80]
Safety assessment	Short-term reactogenicity	Evaluation of immune configuration and signaling balance	Immunopathology may precede overt clinical disease	[2,9,83]
Vaccine platforms	Fixed formulation with limited immune modulation	Platform-specific immune signatures	Vaccine platforms actively shape immune trajectories	[62,84,85]

ADE, Antibody-dependent enhancement.

## Data Availability

Data are contained within the article.
